# Evaluation of the effect of a novel nursing intervention in the treatment of vacuum sealing drainage in patients with chronic orthopedic wounds

**DOI:** 10.4314/ahs.v24i4.13

**Published:** 2024-12

**Authors:** Lan Chen, Dong Li, Xiaodong Lei, Bixia Zhou, Lili Chen

**Affiliations:** Department of Orthopedics, The Third People's Hospital of Chengdu, Chengdu, China

**Keywords:** Orthopedics, chronic wounds, pattern construction, vacuum sealing drainage, wound care

## Abstract

**Background:**

To explore the application effect of “4+1” nursing intervention in the treatment of chronic orthopaedic trauma with negative pressure vacuum sealing drainage.

**Methodology:**

Patients before the implementation of the “4+1 nursing intervention” program were selected as the control group (60 cases). 60 patients after the implementation of the “4+1 nursing intervention” program were selected as the research group. Control group was given routine care. Research group adopted the “4+1” nursing intervention. Hamilton Depression score, wound healing time, pain visual analogue scale, treatment compliance, Self-Rating Anxiety Scale, and nursing satisfaction were compared between the two groups.

**Results:**

After the intervention, the Self-Rating Anxiety Scale and Hamilton Depression scores in the study group were lower than those in the control group; visual analogue scale on the 3rd and 5th days after the study were lower than those in the control group, and the wound healing time was shorter than that in the control group. The treatment compliance rate and nursing satisfaction of the study group were higher than those of the control group (P<0.05).

**Conclusion:**

The application of “4 + 1” nursing intervention in treatment of orthopaedic chronic wounds has a good effect.

## Introduction

Vacuum sealing drainage (VSD) is currently the main clinical treatment method for orthopaedic chronic wounds, as it can effectively improve wound blood circulation, promote wound healing, and reduce the risk of infection^1^,^2^. However, orthopedic chronic wounds have a slow healing rate, requiring long-term treatment that can be painful and expensive, resulting in a significant physical and mental impact on patients and reducing their treatment compliance^3^.

Despite the significant improvement in basic nursing service levels in Chinese hospitals, the refinement of specialist nursing work is still in its infancy, and most hospitals have not yet formed a scientific and refined orthopedic chronic wound nursing architecture. This limitation restricts the improvement of the quality of chronic wound nursing services in hospitals^4^.

To further optimize the effect of orthopedic chronic wound nursing intervention, promote wound healing, and reduce the psychological and economic burden of treatment for patients, we used the “4 + 1” nursing intervention model. This model consists of “in-hospital position nursing, evidence-based psychological intervention, drainage and wound nursing, rehabilitation exercise guidance” combined with “out-of-hospital life guidance” for nursing intervention in orthopedic chronic wound patients receiving VSD treatment. The nursing effect was satisfactory.

## Patients and methods

### Patients

The clinical data of 120 patients with the chronic wound in the Department of Orthopaedics of our hospital from January 2020 to December 2021 were retrospectively analysed. The patients before the implementation of the “4 + 1 nursing intervention” protocol from January 2020 to December 2020 were set as the control group (receiving routine care, 60 patients), and the patients after the implementation of “4 + 1 nursing intervention” protocol from January 2021 to December 2021 were set as the study group (receiving “4 + 1” protocol care, 60 patients). The inclusion criteria for this study were as follows: (1) patients who met the diagnostic criteria for chronic wounds^5^; (2) patients who received VSD hospitalization at our hospital; (3) patients whose clinical data were complete; and (4) patients who were fully informed of VSD treatment and nursing content. The exclusion criteria were as follows: (1) patients with other injuries affecting limb motor function; (2) patients with acute and chronic infections in other locations; and (3) patients with mental or psychological diseases. This study was approved by the ethics committee of The Third People's Hospital of Chengdu. Signed written informed consents were obtained from the patients and/or guardians.

### Methods

All patients received VSD treatment. Patients in the perioperative control group received routine nursing intervention: including health education, life guidance, medication guidance, psychological intervention, complication monitoring, adverse drug reaction monitoring and rehabilitation training guidance, and other basic nursing measures. During hospitalization and before discharge, patients were instructed to adhere to a light diet and monitor their daily energy intake, maintain regular routines, and take precautions to protect the wound surface to prevent wound compression.

The study group received the “4 + 1” nursing program intervention:

(1) **Specialized nursing group:** the head nurse convened the experienced specialist nurse to establish the “4 + 1” nursing group, to facilitate the orderly development and accurate implementation of nursing interventions, trained the nurses in the group, systematically studied the evidence-based nursing measures for orthopedic chronic wounds through literature analysis, case study, and other methods, and collected the actual nursing needs of patients through interviews with clinical patients.

(2) “**In-hospital 4 items” nursing:** 1) Evidence-based psychological intervention: On the day of admission, the nurses used self-rating anxiety scale (SAS) to assess the anxiety of patients, combined with Hamilton Depression Rating Scale (HAMD) assessment results to comprehensively assess the psychological status of patients, according to the SAS and HAMD scores, the negative emotions of patients were divided into four levels: severe (SAS or HAMD ≥ 75 points), severe (65 points ≤ SAS or HAMD < 75 points), general (55 points ≤ SAS or HAMD < 65 points), and good (SAS and HAMD < 55 points), and evidence-based psychological intervention plan was developed according to the grading results, psychological intervention measures mainly included communication, psychological counseling, direct attendant, health education and other four methods, of which the negative emotions were graded as severe and severe patients also used four intervention methods for psychological intervention, with a frequency of 2 times/day, and the patients graded as general and good were only given health education and psychological counseling on the day of admission and 1 day before VSD surgery, without other psychological intervention.

2) **Postural nursing:** After VSD surgery, the group nurse guided the patient to maintain the correct sitting, lying, and stereoscopic position one-on-one according to the patient's wound position, popularized the common symptoms and precautions after VSD surgery to the patient during the guidance process, told the patient not to move at will to avoid traction or compression of the wound, reduced the risk of secondary wound injury, and ensured the VAS drainage effect.

3) **Drainage and wound nursing:** The nurse regularly (3 times/day) checks the dressing status of the patient after VSD surgery, observes whether the dressing is collapsed and bulged and whether the drainage tube is unobstructed. The dressing should be replaced in time if abnormal dressing status was found. If the drainage tube is blocked, heparin sodium solution or normal saline should be used for pipeline cleaning. The wound drug should be replaced regularly according to the doctor's advice. The wound status of the patient should be checked during the dressing change. If there is pus exudation, debridement should be performed in time and the attending physician should be informed.

4) **Rehabilitation exercise guidance:** 3 days after VSD surgery, the patient can be instructed to regularly perform finger range of motion training with the permission of the attending physician. The training movements include wrist, ankle, knee, elbow, and toe joint range of motion exercises as well as slow walking, pacing, and in situ leg raising. The nurse selects the appropriate action for training according to the patient's wound position. The training time is 20 min/per day.

(3) **Out-of-hospital life guidance:** One day before discharge, the group nurse informed the patient of the out-of-hospital follow-up content in advance, and distributed the patient with the rehabilitation guidance manual for the postoperative rehabilitation of VSD in chronic wounds so that the patient could consult the relevant knowledge points after discharge. At the same time, the nurse responsible for the follow-up of the patient followed up with the patient. The follow-up time was 2 months after surgery, and regular follow-up was performed at 1 and 4 weeks at a frequency of 2 times/week. During the follow-up period, the post-hospital health management of the patient with chronic wounds was collected, and the patient's life, diet, routines, and other behaviors that may affect the wound recovery were instructed to improve the patient's self-health management ability.

### Observation indicators

1. **Psychological status assessment:** The SAS^6^ and HAMD^7^ scores before and after intervention were compared between the two groups; the former had a total of 20 items, with a total score of 80 points; the latter had a total of 24 items, with a total score of 81 points, and the scores of the two tables indicated that the more severe the anxiety or depression.

2. **Treatment compliance:** a comprehensive evaluation of the patient's compliance during hospitalization, evaluation criteria^8^: 1) poor compliance: during treatment, the patient experienced resistance, struggle, uncooperation and other non-compliance behaviors, requiring long-term intervention by the family or nursing staff, resulting in delayed implementation of relevant treatment and nursing measures; 2) general compliance: although the patient experienced non-compliance behaviors, the relevant treatment and nursing measures were still completed on time after the intervention by the family or nursing staff; 3) good compliance: although the patient experienced non-compliance behaviors, the patient still cooperated to complete the relevant treatment and nursing measures on his/her own; 4) excellent compliance: the whole process of the patient did not show significant non-compliance behaviors, and all treatment and nursing measures were carried out smoothly; compliance = excellent compliance + good + general.

4. **Clinical indicators:** On the 1^st^, 3^rd^, and 5^th^ day after VSD surgery, the visual analogue scale (VAS) for pain^9^ was used to assess the subjective pain sensation of the patients (the total score was 10 points, and the score was positively correlated with the pain sensation); meanwhile, the wound healing time was compared between the two groups according to the patient's follow-up and return visit.

5. **Satisfaction evaluation:** Before discharge, the patient's satisfaction was collected using the self-made satisfaction questionnaire, which was divided into very satisfied (≥ 85 points), basically satisfied (70 ~ 84 points), and dissatisfied (< 70 points) according to the score of the questionnaire; total satisfaction = very satisfied and satisfied.

### Statistical analysis

Data were analysed using Statistical Product and Service Solutions (SPSS) 22.0 (IBM, Armonk, NY, USA). Independent sample t-test was used for comparison between groups for measurement data obeying normal distribution, and independent sample t-test was used for comparison within groups, all expressed as (x̅±s). Count data were tested by χ^2^ and expressed as a rate (%), P<0.05 indicates a statistical difference.

## Results

### Comparison of baseline data between the two groups

There was no significant difference in age (t = 0.626), course of unhealed wound (t = 0.298), body mass index (t = 0.241), chronic wound type (x^2^ = 0.197), and gender (x^2^ = 0.139) between the two groups (P > 0.05) (Table 1).

**Table 1:** Comparison of baseline data between the two groups

Group	N	Age (years)	Disease course (day)	Body mass index(kg/m^2^)	hronic wound type [n, (%)]			Gender [n, (%)]	
					
				Chronic infected wounds		Chronic ulcer	Other types	Male	Female
Study group	60	30.35 ± 4.15	46.41 ± 5.12	22.35 ± 4.15	26 (43.33)	20 (33.33)	14 (23.33)	37 (61.67)	23 (38.33)
Control group	60	29.89 ± 3.89	46.68 ± 4.80	22.17 ± 4.02	27 (45.00)	21 (35.00)	12 (20.00)	35 (58.33)	25 (41.67)
t/X^2^		0.626	0.298	0.241	0.197			0.139	
P		0.532	0.766	0.810	0.906			0.709	

### Comparison of psychological status between the two groups

After the intervention, the SAS (t = 10.792) and HAMD (t = 11.570) scores of the study group were lower than those of the control group (P < 0.05) (Table 2)

**Table 2:** Comparison of psychological status scores between the two groups (x̅±s, minutes)

Group	N	SAS		HAMD	

		Pre-intervention	Post Intervention	Pre-intervention	Post Intervention
Study group	60	52.35 ± 4.26	15.33 ± 3.59a	45.14 ± 5.30	18.59 ± 2.61a
Control group	60	51.47 ± 4.11	25.52 ± 4.28a	44.83 ± 5.13	27.27 ± 3.59a
t		0.880	10.792	0.249	11.570
P		0.382	< 0.001	0.804	< 0.001

### Comparison of treatment compliance between the two groups

The treatment compliance rate in the study group (95.00% vs. 80.00%) was higher than that in the control group (x^2^ = 5.175, P < 0.05) (Table 3).

**Table 3:** Comparison of treatment compliance between the two groups [n, (%)]

Group	N	Excellent compliance	Good compliance	Fair compliance	Poor compliance	Compliance rate
Study group	60	37 (61.67)	15 (25.00)	5 (8.33)	3 (5.00)	57 (95.00)
Control group	60	30 (50.00)	12 (20.00)	6 (10.00)	12 (20.00)	48 (80.00)
X^2^						4.876
P						0.027

### Comparison of clinical indicators between the two groups

The difference in the VAS on the 1st day after VSD surgery between the two groups had no statistical significance (P > 0.05); the VAS on the 3rd and 5th days after VSD surgery in the study group was lower than that in the control group, and the wound healing time in the control group (P < 0.05) (Table 4).

**Table 4:** Comparison of clinical parameters between the two groups (x̅±s)

Group	N	VAS (points)			Wound healing time (d)

	Day 1 after VSD surgery		Day 3 after VSD surgery	Day 5 post-VSD surgery	
Study group	60	5.47 ± 0.55	4.59 ± 0.93	2.63 ± 0.52	28.75 ± 3.38
Control group	60	5.56 ± 0.62	3.42 ± 1.12	3.79 ± 0.70	34.26 ± 4.05
X^2^		0.841	6.225	10.304	8.091
P		0.402	<0.001	<0.001	<0.001

### Comparison of nursing satisfaction between the two groups

The satisfaction rate of nursing care in the study group (95.00% vs. 81.67%) was higher than that in the control group (x2 = 5.175, P < 0.05) (Table 5).

**Table 5:** Comparison of nursing satisfaction between the two groups [n, (%)]

Group	N	Very satisfied	Basically satisfied	Not Satisfied	Nursing satisfaction rate
Study group	60	37 (61.67)	20 (33.33)	3 (5.00)	57 (95.00)
Control group	60	25 (41.67)	24 (40.00)	11 (18.33)	49 (81.67)
X^2^					5.175
P					0.023

## Discussion

Orthopedic chronic wounds can be caused by various factors, and their treatment may not result in functionally or anatomically intact wounds even after a month10. These types of wounds include chronic infectious wounds, chronic ulcers, and burn wounds, among others. They are generally characterized by local wound ischemia and hypoxia, bacterial biofilm formation, secondary infection, and large wound defects, making them challenging to treat over an extended period^11^. However, Vacuum Sealing Drainage (VSD) can be used to seal orthopedic chronic wounds and provide controllable negative pressure for antibiotic suction and irrigation. This can effectively control wound infection, inhibit bacterial biofilm formation, and promote wound healing^12^.

Unfortunately, the extended healing time required for orthopedic chronic wounds may result in patients experiencing pathological pain and pain caused by frequent dressing changes, reducing treatment compliance. Therefore, standardized nursing intervention during VSD treatment for orthopedic patients with chronic wounds is necessary^13^. Traditional nursing interventions for orthopedic wound patients usually involve basic nursing measures such as health education, life guidance, medication guidance, and conventional psychological intervention. This nursing model process is usually single and rigid, with a high degree of standardization and low refinement. The lack of targeted evidence-based nursing makes it difficult for conventional nursing to achieve satisfactory results, with certain limitations^14^.

In this study, we retrospectively analysed the clinical data of 120 patients with chronic wounds who received treatment at the Department of Orthopaedics in our hospital, using the historical control method. The results showed that the SAS and VAS scores on the 3^rd^ and 5^th^ day after VSD surgery were lower in the study group that received the “4 + 1” nursing intervention model compared to the control group. Additionally, the treatment compliance rate was higher in the study group, indicating that the “4 + 1” nursing intervention had significant advantages in reducing negative emotions of patients with chronic wounds in the Department of Orthopaedics, postoperative pain of VSD, and improving treatment compliance. These findings are similar to a previous study^15^.

The results also indicated that the wound healing time was shorter in the study group, and nursing satisfaction rates were higher compared to the control group. This suggests that the “4 + 1” nursing intervention effectively shortens the wound healing time of patients and improves their satisfaction.

The “4 + 1” nursing intervention model is based on the evidence-based concept and utilizes a group model to concentrate high-quality nursing resources. This nursing intervention model employs literature analysis, case studies, patient interviews, and other methods to collect evidence-based nursing measures and identify nursing needs of orthopedic patients with chronic wounds VSD during treatment. Based on this information, the “4 + 1” nursing architecture was developed, which consists of 4 in-hospital and 1 out-of-hospital nursing interventions, all patient-centered.

The “4 + 1” nursing intervention model effectively reduces the impact of wound compression, wound infection, and negative emotions on the treatment and physical rehabilitation of patients through in-hospital position nursing, evidence-based psychological intervention, drainage and wound care, and other nursing measures. This nursing intervention model promotes the recovery of patients' limb function through rehabilitation exercise guidance and provides guidance for discharged patients through twice-weekly follow-up appointments, including several nursing interventions at the physical and psychological levels, thereby improving treatment compliance and promoting wound healing.

## Conclusion

In summary, the application of “4 + 1” nursing intervention in VSD treatment of orthopedic chronic wounds has a good effect, which can effectively reduce the negative emotions of patients with orthopedic chronic wounds, shorten the wound healing time of patients and improve the treatment compliance and satisfaction of patients. It has the value of clinical promotion.

## Data Availability

The datasets used and analysed during the current study are available from the corresponding author upon reasonable request.
